# Supplementary material for: CCL25/CCR9 interaction promotes the malignant behavior of salivary adenoid cystic carcinoma via the PI3K/AKT signaling pathway
Source: PeerJ. 2022 Aug 19;10:e13844. doi: 10.7717/peerj.13844 (PMC9394511; doi:10.7717/peerj.13844)
Supplement: Supplemental Information 1 [file peerj-10-13844-s001.docx]

**Supplemental Table 1**

| Antibody | Catalogue Number and Manufacturer | | Origin and Dilution | |
| --- | --- | --- | --- | --- |
| anti-ERK1/2 | ab184699 | Abcam, Cambridge, UK | Rabbit | WB (1:1000) |
| anti-pERK1/2  ERK1(T202/Y204)/  ERK2 (T185/Y187) | MAB18251 | R&D Systems, Minnneapolis, MN, USA | Rabbit | WB (1:500) |
| anti-AKT | ab8805 | Abcam, Cambridge, UK | Rabbit | WB (1:1000) |
| anti-pAKT(Ser473) | ab81283 | Abcam, Cambridge, UK | Rabbit | WB (1:1000) |
| anti-STAT3 | ab68153 | Abcam, Cambridge, UK | Rabbit | WB (1:1000) |
| anti-pSTAT3 | 9145S | Cell Signaling Technologies, Danvers, MA, USA | Rabbit | WB (1:500) |
| anti-CCR9 | PA1-21618 | Thermo Fisher Scientific, Waltham, MA, USA | Goat | IHC (1:100) |
| anti-E-cadherin | 3195S | Cell Signaling Technologies, Danvers, MA, USA | Rabbit | IHC (1:200)  WB (1:1000) |
| anti-BCL2 | 12789-1-AP | Proteintech Group, Wuhan, China | Rabbit | WB (1:500) |
| anti-BAX | ab32503 | Abcam, Cambridge, UK | Rabbit | WB (1:1000) |
| anti-caspase 3 | ab4051 | Abcam, Cambridge, UK | Rabbit | WB (1:2000) |
| anti-cyclin D1 | sc-8396 | Santa Cruz Biotechnology, Dallas, TX, USA | Mouse | WB (1:200) |
| anti-c-Myc | ab32072 | Abcam, Cambridge, UK | Rabbit | WB (1:1000) |
| anti-vimentin | ab45939 | Abcam, Cambridge, UK | Rabbit | IHC (1:200)  WB (1:1000) |
| anti-Ki67 | ab279653 | Abcam, Cambridge, UK | Mouse | IHC (1:1000) |
| anti-MMP2 | ab86607 | Abcam, Cambridge, UK | Mouse | WB (1:1000) |
| anti-MMP9 | ab38898 | Abcam, Cambridge, UK | Rabbit | WB (1:1000) |
| anti-SLUG | ab27568 | Abcam, Cambridge, UK | Rabbit | IHC (1:50)  WB (1:1000) |
| anti-SNAIL | ab216347 | Abcam, Cambridge, UK | Rabbit | WB (1:1000) |
| anti-TWIST | ab50887 | Abcam, Cambridge, UK | Mouse | WB (1:1000) |
| anti-GAPDH | 60004-1-Ig | Proteintech Group, Wuhan, China | Mouse | WB:(1:2000) |

**Table S1 Origin and dilution of the primary antibodies**
