# Supplementary material for: CCL25/CCR9 interaction promotes the malignant behavior of salivary adenoid cystic carcinoma via the PI3K/AKT signaling pathway
Source: PeerJ. 2022 Aug 19;10:e13844. doi: 10.7717/peerj.13844 (PMC9394511; doi:10.7717/peerj.13844)
Supplement: Supplemental Information 2 [file peerj-10-13844-s002.docx]

**Supplemental Table 2**

**Table S2 General information for SACC patient samples**

| Clinical Characteristic | Total cases | CCR9 expression | |
| --- | --- | --- | --- |
|  |  | Low | High |
| SACC | 30 | 10 | 20 |
| Age |  |  |  |
| ≤ 45 | 8 | 2 | 6 |
| >45 | 22 | 8 | 14 |
| Gender |  |  |  |
| Male | 21 | 8 | 13 |
| Female | 9 | 2 | 7 |
| Pathological type |  |  |  |
| Cribriform | 10 | 5 | 5 |
| Tubular | 7 | 3 | 4 |
| Solid | 13 | 2 | 11 |
