# Supplementary material for: CCL25/CCR9 interaction promotes the malignant behavior of salivary adenoid cystic carcinoma via the PI3K/AKT signaling pathway
Source: PeerJ. 2022 Aug 19;10:e13844. doi: 10.7717/peerj.13844 (PMC9394511; doi:10.7717/peerj.13844)
Supplement: Supplemental Information 3 [file peerj-10-13844-s003.docx]

**Supplemental Table 3**

**Table S3 Primer sequence of the target genes**

| **Gene** | | Forward Primer 5’-3’ | Reverse Primer 5’-3’ |
| --- | --- | --- | --- |
| **Human**  **origin** | CCR9 | AGAGTGAAGACCATGACCGACA | GGCGGAATCTCTCACCCACAAA |
|  | vimentin | GACGCCATCAACACCGAGTT | GACGCCATCAACACCGAGTT |
|  | MMP2 | AAGTCTGAAGAGCGTGAAGTTTGGA | TGAGGGTTGGTGGGATTGGAG |
|  | MMP9 | AGTCCACCCTTGTGCTCTTCCC | TCTGCCACCCGAGTGTAACCAT |
|  | SLUG | TCTGCCACCCGAGTGTAACCAT | GAGAGGCCATTCGGTAGCTG |
|  | SNAIL1 | CACTATGCCGCGCTCTTTC | CACTATGCCGCGCTCTTTC |
|  | TWIST | GTCCGCAGTCTTACGAGGAG | GCTTGAGGGTCTGAATCTTGCT |
|  | cyclinD1 | GCTTGAGGGTCTGAATCTTGCT | GTCACACTTGATCACTCTGG |
|  | Ki67 | TTTGGGTGCGACTTGACG | GATAGTAACCAGGCGTCTCG |
|  | c-Myc | GTCACACTTGATCACTCTGG | CTTTTCCACAGAAACAACATCG |
|  | BCL-2 | CTTTTCCACAGAAACAACATCG | GAGACAGCCAGGAGAAATCAA |
|  | BAX | TTTGCTTCAGGGTTTCATCCA | GAGACACTCGCTCAGCTTCTTG |
|  | caspase 3 | AACTGGACTGTGGCATTGAG | AACTGGACTGTGGCATTGAG |
|  | GAPDH | CTCCTCCTGTTCGACAGTCAGC | CTCCTCCTGTTCGACAGTCAGC |
| **Mouse**  **origin** | vimentin | AAAGCGTGGCTGCCAAGAA | ACCTGTCTCCGGTACTCGTTTGA |
|  | BCL-2 | CCTGTGGATGACTGAGTACCTG | AGCCAGGAGAAATCAAACAGAGG |
|  | cyclinD1 | CATGACCAGTGTGACTCAAAGCAA | CTCAGACATGGCCCTAAACCTTC |
|  | E-cadherin | GGTCATCAGTGTGCTCACCTCT | GCTGTTGTGCTCAAGCCTTCAC |
|  | MMP2 | AGAACTTCCGATTATCCCATGATGA | TGACAGGTCCCAGTGTTGGTG |
|  | MMP9 | GCCCTGGAACTCACACGACA | TTGGAAACTCACACGCCAGAAG |
|  | SLUG | CTGGCTGCTTCAAGGACACATTAG | TGCAGAAGCGACATTCTGGAG |
|  | GAPDH | CATCACTGCCACCCAGAAGACTG | ATGCCAGTGAGCTTCCCGTTCAG |
